# ASPHER Statement: Planning for Winter 2022-23

**DOI:** 10.3389/phrs.2022.1605394

**Published:** 2022-10-03

**Authors:** John Middleton, Nadav Davidovitch, Henrique Barros, Henrique Lopes, Jose M. Martin Moreno, Amanda J. Mason-Jones, Alison McCallum, John Reid, Ralf Reintjes, Mohamud Sheek-Hussein, Judit Simon, Brian Li Han Wong, Lore Leighton, Robert Otok

**Affiliations:** ^1^ Association of Schools of Public Health in the European Region (ASPHER), Brussels, Belgium; ^2^ School of Public Health, Ben Gurion University of the Negev, Beersheba, Israel; ^3^ Institute of Public Health, University of Porto, Porto, Portugal; ^4^ Unit of Public Health, Institute of Health Sciences, Catolica University, Lisbon, Portugal; ^5^ Department of Preventive Medicine and Public Health, Medical School and INCLIVA, University of Valencia, Valencia, Spain; ^6^ Department of Health Sciences, University of York, York, United Kingdom; ^7^ Centre for Population Health Sciences, Usher Institute, University of Edinburgh, Edinburgh, Scotland; ^8^ Department of Public Health and Wellbeing, University of Chester, Chester, United Kingdom; ^9^ Department of Public Health, Hamburg University of Applied Sciences, Hamburg, Germany; ^10^ Institute of Public Health— College of Medicine and Health Sciences, United Arab Emirates University, Al Ain, United Arab Emirates; ^11^ Department of Health Economics, Center for Public Health, Medical University of Vienna, Vienna, Austria; ^12^ Department of International Health, Care and Public Health Research Institute – CAPHRI, Maastricht University, Maastricht, Netherlands; ^13^ The International Digital Health & AI Research Collaborative (I-DAIR), Geneva, Switzerland

**Keywords:** climate breakdown, Ukraine war, cost of living crisis, COVID-19 pandemic, winter planning

## Introduction

The Covid-19 pandemic has shown European governments were not properly prepared for such a public health emergency affecting all our countries and with deep health, social and economic consequences. Public health and other essential public services had been depleted through years of austerity [1]. This winter the world faces a series of existential threats which require leadership and action from our public health community, advising and supporting national and international efforts to protect and maintain the health of people and the planet.

## The COVID Pandemic is Not Over—But the Circumstances Have Changed

In the last 2 years, ASPHER has set out our recommendations for planning for winter during the continuing pandemic. Such advice, along with that of other learned scientific and public health authorities was followed inadequately by many governments, with the result that new variants of SARS_COV-2 virus, and new surges in cases and deaths occurred [2, 3]. The Covid-19 pandemic is not over, and we will need to continue efforts to control its spread if we are not to see new variants and new threats to health caused by this virus. These issues are covered in detail in an accompanying ASPHER pandemic statement [4].

## Where There is No Vision, the People Perish

This year, the public health situation is not the same as in the previous 2 years; in functional and practical terms it is much worse. The problems we face this autumn and winter reflect further deterioration in the social, political, and economic determinants of health. They also reflect the impact of the hottest summer on record with droughts, wildfires and major floods alternating in many areas in Europe due to the climate crisis [5]. The ongoing pandemic has shown us widening inequalities in health and in economic and social conditions [6]. Socially excluded and marginalised communities have paid an excessive cost. The exhaustion and burn out of workers in key public services and parts of the economy remain common challenges. We are directly and indirectly effected by the fear of war, by the needs of refugees and by impacts on fuel and commodity prices, and by the threats of shortage and disruption of vital services [7]. The cost-of-living crisis is impacting disproportionately on the poorest people in our societies [8]. Uncertainty, the spread of fake or inaccurate news, and changes in technical recommendations that were not always perceived to be effective have undermined confidence in our own public health professionals [9]. Public mental ill-health is now a complex manifestation of climate despair, fear of war, frustration with authorities, information exhaustion, and mass-post traumatic distress disorder consequent on our experiences of the pandemic. There is a collective feeling of powerlessness and hopelessness [10]. The war in Ukraine has led to a reallocation of resources that were originally planned for health, to military development. There has been inadequate will to tackle health, environmental and social problems from our political leaders, a lack of collaboration and solidarity within and between countries, and little vision of what needs to be done. And where there is no vision, the people perish.

## Planning for all Risks and Addressing the Impact of Inequalities

These threats we face are compounding each other. They demand responses from all countries and from the international community. Planning is essential. Governments should apply the mantra of “Hope for the best, but plan for the worst.”

Governments should ensure they have in place comprehensive and integrated planning arrangements which can address multiple security and health risks at the same time: that means being able to recognize and respond to economic, infrastructural and transport risks, food, water, fuel disruptions, social, health and environmental disasters. Failure to strengthen public health systems for current and future challenges, using the tools that are already within our control, as countries and as a WHO region, is unacceptable. Many European countries still have unacceptably low COVID-19 vaccine coverage including gaps in minority groups. Failure to address COVID-19 will mean a failure to protect the economies and vital public services our countries rely on [11].

Governments must have in place strong scientific sources of advice capable of providing expertise on all aspects of these threats to health.

They should also use acknowledged international agency expertise where their own resources may not be sufficiently developed. At local government level, public health systems funding, capability and leadership should also meet high standards.

Governments should plan to address inequalities in the impacts of the various threats to the health and economic wellbeing of poorer, vulnerable, and marginalised groups in their societies.

Schools of public health have the potential to increase capacity building efforts, assist their respective governments, and our international health agencies, in delivering better plans for this winter, to maintain the conditions for protecting health [12].

There is a fundamental challenge for all in our public health systems reflected in the complexity, uncertainty and interacting forces from the continuing pandemic, re-emerging winter viruses, food shortages, fuel poverty and climate driven disasters. Public health leaders will need to draw together expertise and support from many disciplines and systems, such as for surveillance and response evaluation. We need a renewed interest in systems thinking and action. For instance, in 2020 a pre-pandemic analysis of ‘Drivers of change of relevance for Europe’s environment and sustainability’ highlighted six “clusters” of risks and threats [13]. We still face these drivers of change in 2022, amplified by the pandemic and Ukraine war impacts. As winter approaches, we must address the health effects of cold and fuel poverty [14]. In terms of multiple health impacts and shared aetiologies we should return to concepts such as syndemics and co-morbidities [15].

European schools have an ethical and moral obligation towards other regions of the planet, particularly where scientific and economic resources are lacking. We must commit to global solidarity, in the same way as we ask our leaders to do.

ASPHER stands ready to support our member schools, our regional and global partner agencies, and our governments with our expert advice.

## Conclusion: Planning for Prevention, Planning for an Outbreak of Health

We call on our leaders to show their will and commitment to prevent the worst excesses of the health threats we face this winter. We call on them to put in place planning systems and the scientific expertise to address the threats to health faced by all our countries.

The wider public health agenda which our leaders should commit to is an agenda which plans to prevent these problems arising in the first place- it is the agenda of World Health Organization’s “Health for All” principles which address the wider determinants of health: peace, adequate income, safe housing and shelter, clean water and sanitation, adequate food, rewarding work and a satisfying role in society, a safe environment and transport [16]. Policies which have health as a key driver and an explicit goal, will improve health and the environment and reduce the threats we are now faced with this winter. We urge our world leaders to recommit to Health in All Policies [17], and to the Sustainable Development goals. We need an explicit effort to reducing inequalities in health; and we need to protect our planet and fellow species on earth through renewed policies in support of one Health and Planetary Health.

## Author’s Note

The Association of Schools of Public Health in the European Region (ASPHER) is the key independent European organization dedicated to improving and protecting public health by strengthening education and training of public health professionals for practice and research. www.aspher.org.

## Editorial Note

ASPHER is responsible and liable for all contents of ASPHER statements. ASPHER statements are approved by an Editor-in-Chief but not externally peer reviewed.
